# Giant squamous cell carcinoma of the cheek

**DOI:** 10.11604/pamj.2018.31.59.15850

**Published:** 2018-09-27

**Authors:** Youssef Zemmez, Naoufal Hjira

**Affiliations:** 1 Department of Dermatology, Military Hospital Mohamed V, Faculty of Medicine and Pharmacy of Rabat, Rabat, Morocco

**Keywords:** Squamous cell carcinoma, histology, surgery

## Image in medicine

A 60-year-old woman with a history of non-insulin-dependent diabetes reported a case of cutaneous swelling in her right cheek. The swelling had been evolving for 18 months while gradually increasing in size. The dermatologic examination revealed an ulcero-budding lesion, measuring 5cm at large diameter, rounded, with an ulcerated center and a fibrinous base. It was voluminous, sitting on the level of the right cheek pushing the right wing of the nose and causing an occlusion on right eyelid, but the ganglionic areas were free. A skin biopsy was carried out and histology indicated that a moderately differentiated squamous cell carcinoma was present. The patient was referred to the plastic surgery department for surgical removal of the tumor. The cutaneous squamous cell carcinoma is an invasive tumor that develops at the expense of epidermal keratinocytes or mucous membranes in oral, anal or genitals. The major risk factor is the solar exposure.

**Figure 1:**
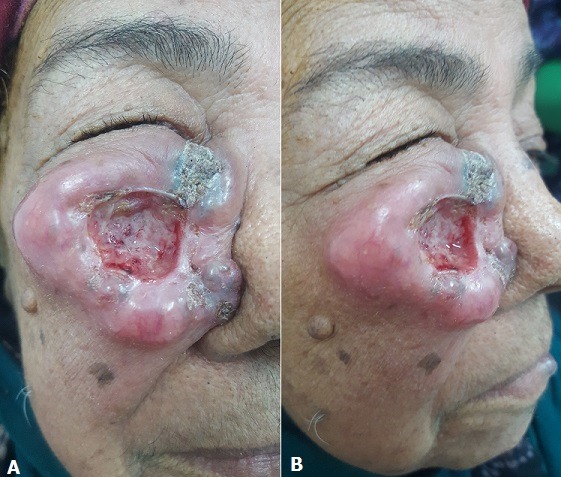
Giant squamous cell carcinoma of the cheek (A,B)

